# Host DNA depletion on frozen human respiratory samples enables successful metagenomic sequencing for microbiome studies

**DOI:** 10.21203/rs.3.rs-3638876/v1

**Published:** 2024-01-23

**Authors:** Minsik Kim, Raymond C. Parrish, Viral S. Shah, Matthew Ross, Juwan Cormier, Aribah Baig, Ching-Ying Huang, Laura Brenner, Isabel Neuringer, Katrine Whiteson, J. Kirk Harris, Amy D. Willis, Peggy S. Lai

**Affiliations:** Division of Pulmonary and Critical Care Medicine, Massachusetts General Hospital; Department of Medicine, Harvard Medical School; Division of Pulmonary and Critical Care Medicine, Massachusetts General Hospital; Division of Pulmonary and Critical Care Medicine, Massachusetts General Hospital; Alkek Center for Metagenomics and Microbiome Research, Baylor College of Medicine; Alkek Center for Metagenomics and Microbiome Research, Baylor College of Medicine; Division of Pulmonary and Critical Care Medicine, Massachusetts General Hospital; College of Science, Northeastern University; Division of Pulmonary and Critical Care Medicine, Massachusetts General Hospital; Division of Pulmonary and Critical Care Medicine, Massachusetts General Hospital; Department of Medicine, Harvard Medical School; Division of Pulmonary and Critical Care Medicine, Massachusetts General Hospital; Department of Molecular Biology & Biochemistry, University of California; Department of Pediatrics, University of Colorado Anschutz Medical Campus; Department of Biostatistics, University of Washington School of Public Health; Division of Pulmonary and Critical Care Medicine, Massachusetts General Hospital; Department of Medicine, Harvard Medical School

**Keywords:** Microbiome, Respiratory, Shotgun metagenomics sequencing, host DNA depletion, low biomass

## Abstract

**Background:**

Most respiratory microbiome studies have focused on amplicon rather than metagenomics sequencing due to high host DNA content. We evaluated efficacy of five host DNA depletion methods on previously frozen human bronchoalveolar lavage (BAL), nasal swabs, and sputum prior to metagenomic sequencing.

**Results:**

Median sequencing depth was 76.4 million reads per sample. Untreated nasal, sputum and BAL samples had 94.1%, 99.2%, and 99.7% host-reads. The effect of host depletion differed by sample type. Most treatment methods increased microbial reads, species richness and predicted functional richness; the increase in species and predicted functional richness was mediated by higher effective sequencing depth. For BAL and nasal samples, most methods did not change Morisita-Horn dissimilarity suggesting limited bias introduced by host depletion.

**Conclusions:**

Metagenomics sequencing without host depletion will underestimate microbial diversity of most respiratory samples due to shallow effective sequencing depth and is not recommended. Optimal host depletion methods vary by sample type.

## INTRODUCTION

The respiratory microbiome has been associated with the development or exacerbation of a broad range of lung diseases ranging from respiratory infections, chronic lung diseases such as asthma, chronic obstructive pulmonary disease, and lung cancer [1]. However, a major barrier to progress is the high host DNA content of many respiratory samples, leading the respiratory microbiome field to rely on amplicon sequencing targeting the small subunit ribosomal RNA (SSU-rRNA) gene regions (typically 16S rRNA) to describe respiratory microbial communities. While SSU-rRNA profiling is less costly and not limited by host DNA content, it has limitations compared to metagenomic next-generation sequencing (mNGS). Each domain requires different regions (bacterial 16S, archaeal 16S, eukaryotic 18S, and ITS) to be amplified and sequenced. Common targets of 16S rRNA short reads such as the V3-V4 regions can identify only taxonomy at the genus level [2, 3]. Untargeted mNGS addresses some of these limitations of amplicon sequencing including cross-domain characterization of microbial communities and the ability to identify microbes at the species or strain level [4], a degree of taxonomic resolution critical for the design of future microbiome-targeted interventions [5]. mNGS additionally can assess predicted functional profiles [6, 7], which is important given the ability of multiple microbes to perform the same community function [8].

During mNGS all DNA, including both mammalian host and microbial DNA, is sequenced. While this is not a problem in certain sample types such as stool which typically has less than 0.5% host DNA content [9], other sample types such as vacuumed dust and skin swabs have on average 50% host DNA [9, 10]. One of the most challenging biospecimens is respiratory samples; with saliva and nasopharyngeal swabs averaging 90% and >99.9% host DNA[10] respectively. A proposed solution for mNGS of samples with high host content has been deeper sequencing, which may be tractable for samples with less than 90% host content. However, for biospecimens with >99% host DNA content, even ultra-deep sequencing is unlikely to overcome the challenges of undersampling due to inadequate effective sequencing depth after host read removal [11]. Other proposed solutions include culture enrichment to increase microbial load prior to mNGS [12] though abundance estimates no longer reflect *in vivo* conditions after culture. Some artificial sputum medium recipes also contain salmon sperm DNA[13], which will also be sequenced with mNGS and may overwhelm microbial-derived reads.

An alternate strategy to address the challenges of mNGS for low-biomass and high-host content samples is selective degradation or binding of human DNA[14, 15]. For example, osmotic lysis followed by propidium monoazide treatment to cross-link free DNA (lyPMA) has been employed for cryopreserved saliva [10]. A benzonase-based approach has been tailored for sputum [16] and later for skin swabs and saliva [17]. Commercial kits have also been developed and tested in tissue specimens [18, 19] and nasopharyngeal aspirate [20]. These studies focused on either treatment of never-frozen samples that required immediate processing at time of sample collection, or samples frozen with cryoprotectants [10]. Host depletion at time of sample collection is resource-intensive and the requirement for cryopreservation before freezing limits the generalizability of these tested methods as most longstanding cohort studies with biorepositories have not added cryoprotectants to respiratory specimens. Some respiratory specimens such as human sputum have natural cryoprotectant properties [21] suggesting that optimal host DNA depletion approaches may differ based on the underlying sample matrix. A head-to-head comparison of host DNA depletion approaches for metagenomics across diverse non-cryopreserved frozen respiratory samples has not been performed.

To address these challenges to the respiratory microbiome field, we evaluated the efficacy of 5 different commonly used methods for host depletion before mNGS using previously frozen whole bronchoalveolar lavage fluid (BAL), nasal swabs, and spontaneously expectorated sputum collected from ongoing human observational studies. Host depletion efficiency was evaluated based on sequencing failure rate, host DNA proportion, final non-human reads, species richness, potential bias compared to the untreated community, and presence of contamination.

## RESULTS

### Host depletion efficiency

Summary statistics describing the effect of host depletion on human and bacterial DNA quantified by qPCR, library preparation and sequencing failure rates, proportion of host mapped reads, effective sequencing depth (final reads after human read removal), species and predicted microbial functional richness are summarized in Table 1. Results of statistical models to assess the effects of host depletion are summarized in Table 2. Based on qPCR, most host depletion methods decreased both total host and bacterial DNA for all sample types (Fig. S1). 13 samples failed library prep based on fragment analysis but were nevertheless still sequenced for further analyses. Four MolYsis, four lyPMA, and two HostZERO treated nasal samples failed library prep. MolYsis, HostZERO, and lyPMA each failed library prep for one BAL sample.

The median sequencing depth of all respiratory samples was 76.4 [interquartile range 46–138.8] million reads. After removal of host reads, untreated samples had a median 0.33, 4.82, and 0.60 million reads for BAL, nasal, and sputum, respectively. Two BAL samples, one untreated and one lyPMA treated, had no microbial mapped reads (Fig. S2) and were considered failed sequencing.

Host DNA content was 99.7%, 94.1%, and 99.2% for BAL, nasal, and sputum samples respectively based on mNGS. Overall, the proportion of host DNA decreased after host depletion treatment (Fig. S3 B, D) though treatment was more effective for nasal and sputum compared to BAL samples. The proportion of host DNA estimated by sequencing and by qPCR were highly correlated (R^2^ = 0.92, Fig. S4A) and had high agreement (Bland-Altman plot Fig. S4B) indicating that qPCR using the primers tested can be used to reliably estimate host DNA content prior to mNGS. Change in % host DNA differed by sample type and host depletion treatment (Table S1). For BAL, the most effective treatment was HostZERO which decreased host DNA proportion by 18.3 [5.6–30.9]%, followed by MolYsis (17.7 [5.1–30.3]%). For nasal, all treatments besides benzonase led to significant differences in % host content with the most effective methods being QIAamp (75.4 [54.0–96.9]% decrease) and HostZERO (73.6 [52.1–94.9]% decrease). For sputum, the most efficient methods were MolYsis (69.6 [58.0–81.3]% decrease) and HostZERO (45.5 [33.8–57.1]% decrease).

Most host depletion treatments significantly increased final reads after host read removal though efficacy differed by sample type (Table S2). Untreated BAL had 0.3 million final reads which significantly increased after all treatments except lyPMA. Specifically, all commercial kits resulted in 10-fold increases in final reads. For nasal swabs, QIAamp increased final reads by 13-fold and HostZERO by 8-fold. For sputum, all treatments increased final reads although MolYsis, HostZERO, and QIAamp had the largest effect sizes, increasing final reads by 100-fold, 50-fold, and 25-fold respectively.

### Host depletion increases observed microbial species and predicted functional richness by increasing effective sequencing depth

**Figure 2:**
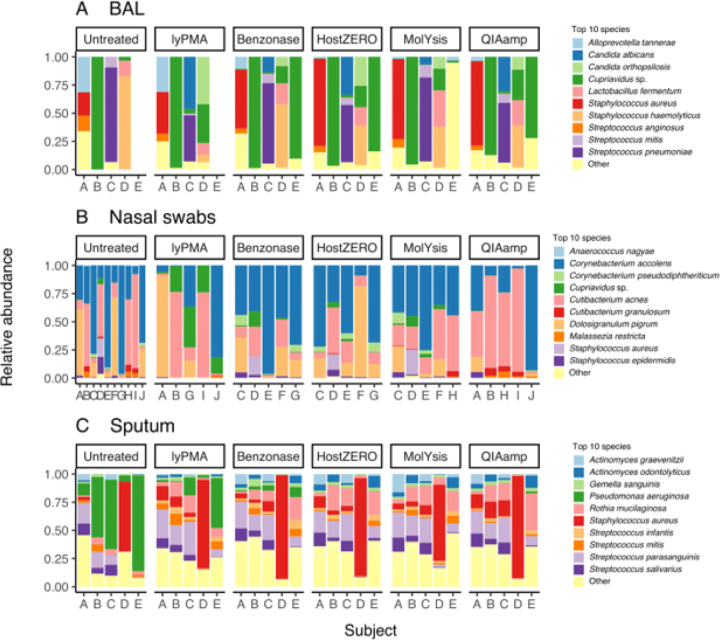
Sample relative read abundances at the species level stratified by sample type and host depletion method. **2A** Broncho-alveolar lavage (BAL) from critically ill patients. **2B**Nasal swab samples from healthy adults. 2C Spontaneously expectorated sputum from people living with cystic fibrosis. Empty space indicates samples that failed sequencing (no microbial reads identified).

**Figure 3:**
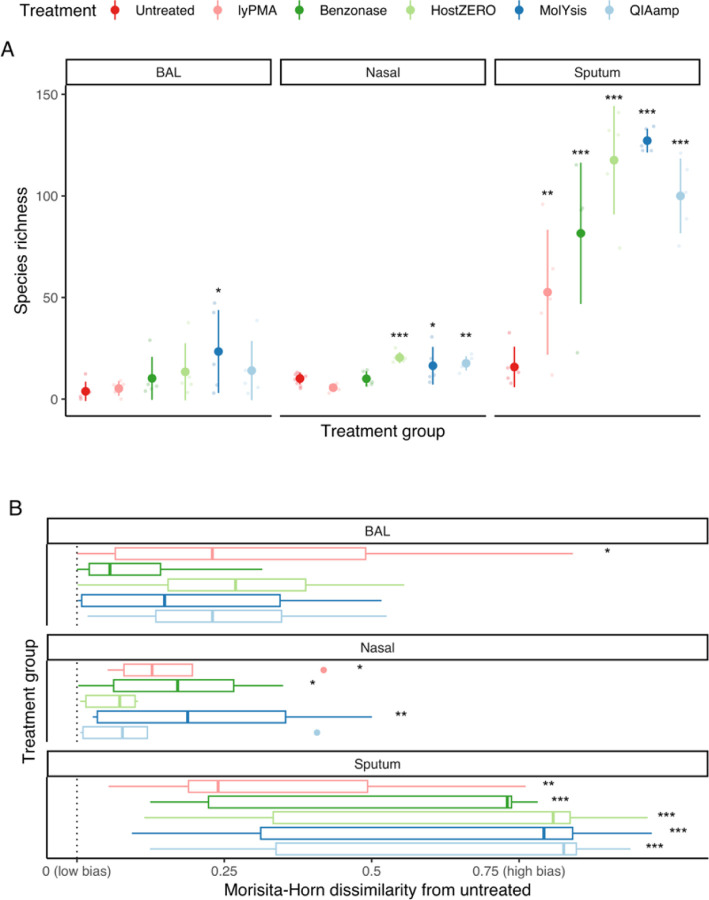
Alpha and beta diversity stratified by sample type and treatment method. **3A**.Species richness in mean values ± SD. **3B**. Boxplot of potential bias measured by Morisita-Horn dissimilarity (1 – MH) between each host depletion method and corresponding untreated sample. Statistical significances were tested with linear mixed effect model adjusting for repeated measures in a participant as a random effect variable. *: *p*-value < 0.05, **: *p*-value < 0.01 and ***: *p*-value < 0.001.

Host depletion leads to a higher effective sequencing depth (final non-human reads), and thus we evaluated the effect of host depletion on observed species richness. Species-level microbial community profiles for untreated and treated samples are depicted in Figure 2. Overall, species richness increased after host depletion (Table 1) although the magnitude of increase differed based on sample type and treatment (Table S3 and Figure 3A). For BAL, only MolYsis showed significantly increased species richness compared to the untreated samples (by 18 [7–30] species). For nasal, HostZERO, QIAamp, and MolYsis increased species richness by 10, 8, and 6 respectively. All host depletion treatments significantly increased the species richness of sputum, with the largest effect sizes being from MolYsis, HostZERO, and QIAamp, with an increase of 111, 102, and 84 respectively.

To determine whether higher final non-human reads explain the increase in species richness after host depletion, we performed a causal mediation analysis with host depletion method, final reads, and species richness as the exposure, mediator, and outcome, respectively (Table S4). Besides lyPMA, all the treatments showed a significant indirect effect. The proportion mediated by HostZERO, MolYsis, and QIAamp was over 50% of the total effect, indicating that the increase in species richness after host depletion was largely explained by the increase in final reads. Similar results were seen when evaluating predicted microbial functional richness.

### Potential bias in microbial community composition due to host depletion treatment

Most host depletion methods rely on the observation that host cells are more vulnerable to lysis than microbial cells. However, gram-negative bacteria are also more vulnerable to treatment effects compared to gram-positive bacteria or fungi. Thus we evaluated the effect of host depletion on the relative abundance of gram-negative bacteria present in a mock community preserved in DNA/RNA Shield (Zymo) (Figure S5) as well as in respiratory samples (Figure S6, Table S5). In analyses stratified by sample type, the effect was the strongest in the mock community compared to respiratory samples, which was expected as the mock community we used is stored in DNA/RNA Shield, a common nucleic acid stabilizing agent contains a mild detergent to inactivate infectious agents and prevent further microbial growth. Host depletion treatment did not decrease the relative abundance of gram-negative bacteria in BAL, and only lyPMA decreased the relative abundance of gram-negative bacteria in nasal. All host depletion treatments decreased the relative abundance of gram-negative bacteria in sputum; note that all sputum samples were obtained from patients with cystic fibrosis. Key members of the cystic fibrosis airway community such as *Pseudomonas aeruginosa* are known to produce large amounts of extracellular DNA [22] and most host depletion protocols rely on removal of extracellular DNA after lysis of host cells.

Changes in overall microbial community structure were analyzed using Morisita-Horn dissimilarity (Figure S7). PERMANOVA analysis was conducted (Table S6) and all treatments showed sample-type-specific effects. To better quantify potential bias from host depletion treatment, we calculated Morisita-Horn dissimilarities between paired samples (untreated and host-depleted) from the same subject and sample type (Figure 3B, Table S7) and used this continuous measure as an outcome in linear mixed effects models. No treatment changed the paired Morisita-Horn dissimilarities for BAL. lyPMA, benzonase, and MolYsis changed the paired Morisita-Horn dissimilarities of nasal. All the treatments changed the paired Morisita-Horn dissimilarities of sputum.

### Effect of host depletion treatment on differential abundance of microbial species

**Figure 4:**
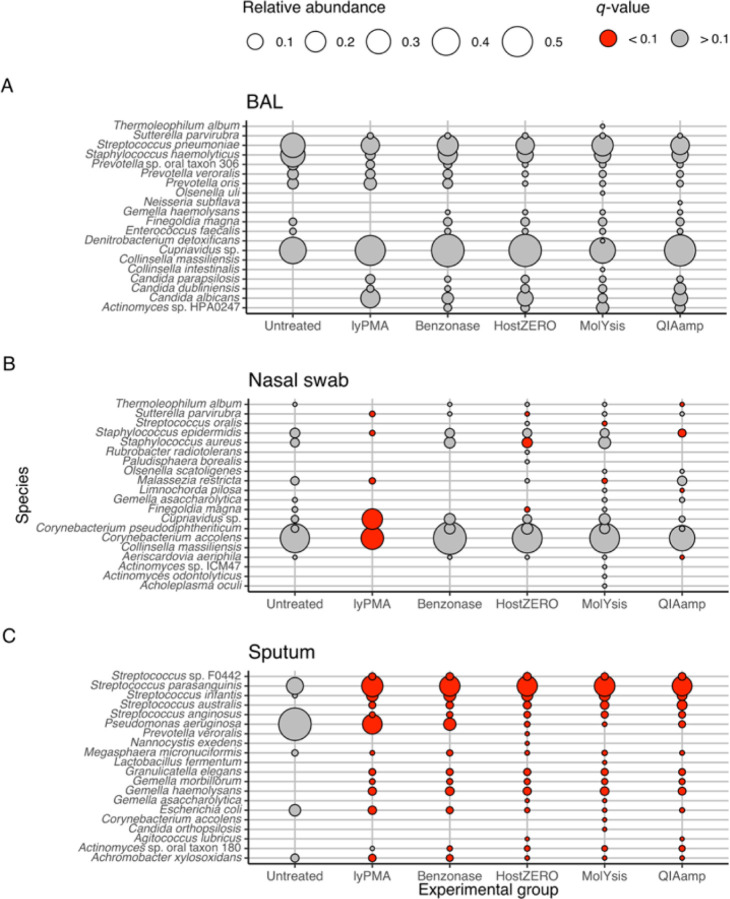
Mean relative abundance of top 20 significant taxa identified by differential abundance analysis using linear mixed effect model (feature ~ lyPMA + Benzonase + HostZero + MolYsis + QIAamp + (1|subject id)) after centered log-ratio transformation. Analyses were stratified by sample type. (A) Bronchoalveolar lavage, (B) Nasal, and (C) Sputum. Statistical significances were noted at the level of *q*-value < 0.1.

To determine whether there are species-specific effects of host depletion treatment, we conducted differential abundance analysis using linear mixed effect models after centered log-ratio transformation, accounting for the fixed effects of sample type and treatment and considering the random effect of each subject (Table S8). Given that most significant associations were due to sample type (Fig. S8), we then performed differential abundance analyses stratified by sample type (Figure 4, top 20 species by minimum *q*-value). For BAL, treatment did not lead to differentially abundant taxa. For nasal samples, 19 taxa were differentially abundant at a significant level of *q* < 0.1. For sputum samples, 111, 102, 101, 86, 82 taxa were differentially abundant compared to untreated sputum samples for QIAamp, MolYsis, HostZero, Benzonase, and lyPMA treatments, respectively.

### Effect of host depletion treatment on predicted microbial community function

Similar to species richness, host depletion increased the richness of predicted microbial community functions (Table 1). Most of the treatments significantly increased predicted functional richness (Figure S9A) and Table S9). For BAL, MolYsis, HostZERO, QIAamp, and benzonase treatment increased functional richness by 190, 165, 130, and 126 pathways respectively. For nasal, HostZERO, QIAamp, and MolYsis increased functional richness by 66, 66, and 54 pathways respectively. For sputum, all treatments increased functional richness, with the largest effect size seen with MolYsis (142), HostZERO (137), and QIAamp (116). Compared to taxonomic profiles, Morisita-Horn dissimilarities in functional profiles showed smaller changes after treatment for nasal and sputum but higher for BAL (Figure S9B). Larger numbers of predicted functions were differently abundant in CPM (copies per million) with differences based on sample type (Figure S10). For BAL, pathways unable to be identified in untreated BAL were detected after most host depletion treatments (Figure S11).

### Sensitivity analysis for potential effect of contamination

Given that increasing effective sequencing depth was associated with increased species and predicted microbial richness (Figure S12), we performed a sensitivity analysis to ensure that the increased species richness was not due to the introduction of contaminants given the low biomass nature of most respiratory samples. We identified potential contaminants using two approaches: the approach implemented in the *decontam* R package [23] and the approach implemented in the t∈yvamp R package [24] (Figure S13). Even after removing the 7 species identified as potential contaminants by decontam (Table S10), and analyzing corrected relative abundances estimated by tinyvamp, host depletion treatment increased species richness. Using mixed effects models stratified by sample type on these decontaminated datasets, the increase in species richness from host depletion treatment remained significant (Table S11).

## DISCUSSION

Respiratory samples have host DNA content often exceeding 95%, making successful characterization of the respiratory microbiome using mNGS challenging even with deeper sequencing due to unobserved richness. We tested five host depletion approaches using published methods or commercial kits and showed that even in previously frozen respiratory samples, significant depletion of host DNA can be achieved. The increase in effective sequencing depth rather than contamination introduced by host depletion treatment explains the observed increase in species richness after host depletion. We saw similar findings when evaluating predicted microbial functional richness. For BAL and nasal samples, most host depletion methods contributed limited bias to microbial community composition. For sputum samples, host depletion changed microbial community composition although it is unclear the extent to which this can be explained by the production of extracellular DNA by members of the CF airway microbiome. Optimal methods for host depletion vary by sample type. Metagenomics sequencing without host depletion will underestimate microbial diversity of most respiratory samples due to shallow effective sequencing depth after host read removal and is not recommended.

Metagenomics sequencing of respiratory samples has high failure rates due to high host DNA content. For example, a study using nasopharyngeal swabs for COVID-19 testing found that 54.7% samples had 100% human reads in mNGS without host DNA depletion [25]. Several studies have evaluated the efficacy of host depletion approaches on respiratory samples for mNGS, though they focused on a single type of respiratory sample, largely evaluated methods using fresh-unfrozen samples, and sequencing depth was significantly lower (32 million reads per sample or less) compared to our study. Marotz et al [10] developed the lyPMA method and found it superior to MolYsis and QIAamp when tested on unfrozen saliva samples. Saliva contains high host DNA content but also has higher microbial load than respiratory samples from patients without infection [26]. When testing frozen non-cryopreserved saliva samples, lyPMA was less effective at reducing host content with more variability in efficacy. Nelson et al. [16] developed the benzonase method and found it superior to an alternate benzonase-based method, lyPMA, and the MolYsis Basic kit designed for small sample volumes of 0.2mL or less (we tested the MolYsis Basic 5, which is designed for sample volumes up to 5 mL). Testing was performed on sputum frozen without a cryopreservative from children with cystic fibrosis; note sputum from patients with chronic infection often have paradoxically also higher host DNA content due to the influx of inflammatory immune cells. Benzonase led to a greater reduction in % host DNA than other methods, whereas we found that the MolYsis Basic 5 kit, followed by HostZERO and QIAamp were the most efficient. Similar to our results, they also noted a decrease in the relative abundance of certain gram-negative bacteria such as *Pseudomonas* and *Achromobacter* in benzonase-treated samples compared to untreated samples. Based on viability studies using culture, benzonase treatment did not decrease the viability of these gram-negative bacteria. Thus investigators concluded that the reduction in gram-negative bacteria was due to removal of extracellular DNA [16]. Rajar et al [27] evaluated frozen nasopharyngeal aspirates cryopreserved with 20% glycerol though their study design included combinations of different host depletion and extraction protocols (including spin column-based protocols which lead to high sample loss) thus limiting interpretability. QIAamp-based host depletion was extracted with a spin column leading to insufficient nucleic acids for sequencing. They found that MolYsis performed the best in combination with an extraction protocol that did not use spin columns. Many ongoing large epidemiological studies have not cryopreserved banked respiratory specimens, nor is host depletion on freshly collected samples before freezing logistically possible as it requires additional trained personnel, equipment, and processing time compared to standard biobanking. Our study focused on non-cryopreserved previously frozen BAL, nasal, and sputum samples and demonstrate that effective host depletion methods exist for each sample type. These results will inform investigators with existing biobanks of respiratory samples on optimal host depletion approaches to perform successful metagenomics sequencing.

Our study has several strengths. We evaluated several different types of respiratory samples and showed that optimal methods based on one sample type cannot necessarily be extrapolated to another sample type. We focused on non-cryopreserved samples, which is more generalizable to most respiratory sample collection methods for existing clinical studies. We performed deep metagenomics sequencing, 76.4 million reads per sample, which is approximately twice that of existing respiratory metagenomics studies. We show that even at this depth, without host depletion there is inadequate characterization of respiratory microbial communities. We used mediation analysis to show that the deeper effective sequencing depth resulting from host depletion explains the majority of effect of host depletion on increased species richness. We performed careful sensitivity analyses to evaluate the potential contribution of contamination and show that even after removal of potential contaminants, host depletion methods increased species richness. Nevertheless, there are some limitations to our study. Our mock community was preserved in DNA/RNA shield, which contains a mild detergent that lyses microbial cells. The manufacturer (Zymo) does not recommend host depletion on samples preserved in DNA/RNA shield for this reason, however we chose this mock community because many ongoing respiratory microbiome studies, particularly since the COVID-19 pandemic, collected respiratory samples in DNA/RNA shield. Thus we felt it was important to demonstrate the degree to which samples collected in this fashion would bias sequencing results with under-representation of gram-negative bacteria. To better assess for bias, untreated BAL, nasal, and sputum samples could have been sequenced much deeper than host-depleted samples to achieve the same effective sequencing depth after host read removal.

In summary, we show that host depletion treatment enables characterization of the respiratory microbiome with mNGS, even in previously frozen samples. Metagenomics sequencing without host depletion will underestimate microbial diversity of most respiratory samples due to shallow effective sequencing depth and is not recommended. Optimal host depletion methods vary by sample type.

## METHODS

### Sample collection

Anterior nasal swab samples were obtained from healthy adults according to a standardized protocol as previously described in an earlier study [28]. PBS was added (1 mL) to the nasal swab samples and vortexed briefly. Four aliquots were made with one nasal swab sample, and a swab and 200 uL of sample solution was utilized for each aliquot. Sputum was collected from adult patients with cystic fibrosis described in a previous study [29, 30]. Briefly, adult persons over age 18 satisfying cystic fibrosis clinical diagnostic criteria and receiving routine care at the Massachusetts General Hospital Adult Cystic Fibrosis Center were recruited. The volume of sputum samples was supplemented with PBS to make 1mL of 6 aliquots, and gently homogenized by syringes to make evenly distributed aliquots. Bronchoalveolar lavage (BAL) fluid was collected from intubated patients for clinically indicated bronchoscopies with excess BAL. For BAL, each sample was evenly separated into 6 individual aliquots to have volume of 200 uL. Ethical approval for this study was obtained by the Institutional Review Board of Mass General Brigham (Protocol #2018P002934, 2019P002868 and 2020P001761).

### Host DNA depletion treatments

5 different host DNA depletion methods (lyPMA, Benzonase, HostZERO, MolYSIS, and QIAamp) were tested for nasal swab, sputum and BAL samples. The total number of treatment groups per each sample was 6 (5 different treatment and 1 control group). Nasal swab samples had 2 control groups due to the ability to collect only 4 nasal swabs per participant at any given time.

For lyPMA, the procedure followed a previously published protocol by Marotz et al [10]. Briefly, samples were centrifuged to collect cells (10,000 g, 8 min). After carefully discarding the supernatant, the pellets were resuspended with 200 ul of DNAse free-water (163049500, Qiagen, Germany) and mixed by Voltex-Gini2. Samples were left at room temperature for 5 min and 5 uL of PMA (40019, Biotium, USA) was added to the sample (10 uL of 1 mM PMA). After briefly vortexing, samples were incubated in the dark room at room temp (5 min). To bind PMA dyes to DNA, samples were placed horizontally on an orbital shaker and exposed to a light source with 2610 lumens (LED A21, GE, USA) at 20 cm distance for 30 min, and rotated every 5 min.

Benzonase treatment method was conducted as described at Nelson et al. [16]. Firstly, 7 mL of DNAse-free water was added to 200 uL sample, and then the samples were placed on an orbital shaker for 1 hour at 60 RPM to lyse mammalian cells. 800 uL of 10x Benzonase buffer (200 mM Tris-HCl (15567027, Invitrogen, USA), 10 mM MgCl2 (AM9530G, Invitrogen, USA)) and 250U of Benzonase (E1014–25KU, Sigma, USA) to each sample (1 uL) was added to the samples, and the mixtures were incubated for 2 hours at 37 C (120 rpm) in an incubator (New Brunswick Innova 42, Eppendorf, Germany). After centrifuging at 8,000 g for 10 min the pellets were resuspend with 1mL PBS and moved to 1mL tubes. The second centrifuge was conducted at 13000g for 3min, the supernatants were removed, and the pellets were resuspended with 400 uL of TE (AM9849, Invitrogen, USA) + 5 mM EDTA (15575–020, Invitrogen, USA).

HostZERO was implemented according to the manufacturer’s protocol (https:/files.zymoresearch.com/protocols/d4310_hostzero_microbial_dna_kit.pdf). Briefly, 1 mL of host DNA depletion solution (D4310–1-20, Zymo, USA) was added per 200 mL of sample. The mixture was agitated by orbital shaking for 15 min at room temp at 180 rpm. After centrifuging the tube at 10,000 g for 5 min at room temperature, the supernatant was carefully removed. 100 uL of microbial selection buffer D4310–2-5, Zymo, USA) and 1 uL of microbial selection enzyme (D-4310–3-50, Zymo, USA) was added to the samples, and the samples were incubated at 600 rpm, 37C for 30 min in a thermomixer.

MolYsis Basic 5 (D301–050, Molzyme, Germany) was implemented following the manufacturer’s protocol. Briefly, 250 uL buffer CM was added to the samples and they were agitated by vortexing for 15 seconds and incubated at room temperature for 5 minutes. Reagents (250 uL buffer DB1, 10 uL MolDNase B) were added to the samples and briefly mixed by vortexing for 15 seconds. After an incubation process at room temp for 15 minutes, samples were centrifuged 12,000 g for 10 minutes and the supernatant was removed carefully. The pellet was resuspended with 1 mL buffer RS and the Vortex Gini2, and centrifuged at 12,000 g for 5 minutes. Finally, 80 uL buffer RL was added to the pellets and mixed with pellets by Vortex Gini.

For QIAamp, the procedure followed the manufacturer’s protocol (https://www.qiagen.com/us/resources/resourcedetail?id=c403392b-0706-45ac-aa2e-4a75acd21006&lang=en). Briefly, after adding 800 uL of PBS (MRGF-6230, Growcells, USA) to each sample to make the total reaction volume 1 mL, 500 uL Buffer AHL (1080302, Qiagen, Germany) was added to 1 mL of sample. Samples were incubated at room temperature for 30 minutes at 600 rpm. The pellet was collected by centrifuging the tube at 10,000 g for 10 minutes and removing the supernatant carefully. After adding 190 uL of Buffer RDD (1018702, Qiagen, Germany) and 2.5 uL of bezonase (1038893, Qiagen, Germany), the samples were incubated at 37C for 30 minutes at 600 rpm. 20 uL of Proteinase K was added and samples were incubated at 56C for 30 minutes at 600 rpm afterwards. All incubation processes were conducted with a thermomixer (Thermomixer C 5382, Eppendorf, Germany).

### DNA extraction

The same nucleic acid extraction approach was applied to all sample types as previously described [31]. In brief, treated and untreated samples, reagent-only negative controls, and mock community positive controls (Zymo Research) were extracted using a protocol optimized for respiratory samples with a magnetic bead-based protocol using the Maxwell HT 96 gDNA Blood Isolation System (Promega) on a KingFisher Flex instrument. Briefly, cetyl trimethyl ammonium bromide (CTAB) is added to samples in individual Lysing Matrix E tubes (MP Biomedicals), incubated at 95°C for 5 minutes followed by beadbeating for three 30 second cycles at 7.0 m/s, incubated with proteinase K at 70°C for 10 minutes, 300 sample µL lysate collected, additional beadbeating for three 30 second cycles at 7.0 m/s with each cycle, and additional 300 sample µL lysate collected. Sample lysates are transferred to 96 well plates for binding, washing, and elution steps on the Kingfisher Flex sample purification system.

### Quantitative polymerase reaction (qPCR)

Quantification of human DNA was determined focusing on the LINE-1 region with the Femto human DNA quantification kit (Zymo E2005, USA) with standards. Bacterial DNAs were measured targeting 16S region with a set of universal primers (5’-CCTACGGGAGGCAGCAG-3′ and 5’-ATTACCGCGGCTGCTGG-3′) for bacterial 16S rRNA^19^ and bacterial DNA standards (Zymo E2006–2, USA) for quantification. All reactions were performed in triplicate. Absolute quantification was determined using standard curves generated according to the manufacturer’s protocol (https://files.zymoresearch.com/protocols/_e2006_femto_bacterial_dna_quantification_kit_ver.pdf and https://files.zymoresearch.com/protocols/_e2005_femto_human_dna_quantification_kit.pdf).

### Metagenomics sequencing and data processing

PicoGreen dsDNA assay kits were used for DNA concentration measurement at library preparation (P7589, Invitrogen, USA). Due to low microbial DNA content, a DNA library prep kit (E6177L, New England Biolabs, USA) designed for high range of input (100–500 ng) was used. For all sample types, 1:25 diluted adapter was used during adapter ligation, and 12 cycles of PCR amplification was conducted. Success of library preparation was assessed with fragment analyzer (DNF-474–0500, Agilent, USA) and qubit (Invitrogen, USA). In total 157 samples (30 BAL, 35 nasal, 30 sputum, 30 negative control from host depletion, 30 positive controls from host depletion, 1 extraction positive control, and 1 extraction negative control) were sequenced on the Illumina NovaSeq platform targeting 10 Gb/sample. Reads were processed with Casava (Illumina) and bbduk to retrieve sequences and remove Illumina adapters.

Profiling of metagenomes were processed with bioBakery 3 [7] combined with bowtie2 with hg38 reference database for mapping and removing human reads [32]. Specifically, MetaPhlAn 3.0 and HUMAnN 3 were employed for taxonomical and functional profiling, respectively. Community profiles, either microbial taxonomy or predicted function of genes, and their hierarchical structures were merged by *phyloseq* package v1.41.1 [33]. Outputs were normalized to relative abundances considering the length of core genomes used for the identification for taxa, and reads per kilo million for function, respectively. Proportions of host DNAs in a sample was calculated using both qPCR and mNGS results using the following equations.

eqn. 1 $$\text{Host DNA }\left(\text{qPCR}\right)\;\left[\%\right]=H_{q}/\left(B_{q}+H_{q}\right)$$

eqn. 2 $$\text{Host DNA }\left(\text{sequencing}\right)\;\left[\%\right]=R_{H}/\left(R_{C}\right)$$

Where, $H_{q}$ is the absolute amount of host DNA quantified by qPCR with LINE-1 region, and $B_{q}$ is the amount bacterial DNA quantified by qPCR with 16S region, $R_{H}$ is the host reads identified by bowtie2 among $R_{C}$, and the $R_{C}$ is the cleaned reads after removing low quality reads. Furthermore, low prevalent taxa were removed at a 5% threshold for statistical analyses, to avoid resulting in wrong association i.e. detecting more taxa after host DNA depletion [34].

### Statistical analyses

Statistical analyses were pre-registered on the Open Science Foundation (https://osf.io/2jtc5) [35]. All statistical analyses were conducted in R version 4.3.1 (https://www.r-project.org). Library preparation success rates were assessed with a logistic mixed-effect model using the glmer function from *lme*4 R package v1.1.34 [36]. Final reads, % host DNA and other continuous outcomes were assessed by linear mixed effect models using lme4::lmer function. Predictors of the models were sample type, treatment method, and an interaction term for sample type x treatment method. Repeated measurements from one participant were accounted for with a random effect term. Alpha diversity indices were calculated with *vegan* package v2.6.4 [37]. Beta diversity was calculated with the vegan::vegdist, where Morisita-Horn dissimilarity index was calculated by subtracting the Morisita-Horn similarity index from 1, ordinated with the *phyloseq*, and paired distances extracted using ↔ *ietr* package v0.2.3 (https://github.com/andersgs/harrietr). Stratified analyses by sample type were conducted when the sample type * treatment method interaction term was significant. Predictors of overall microbial community structure was evaluated on beta diversity using permutational analysis of variance (PERMANOVA). All the predictors were tested as fixed effects to compare the effect size between them (feature ~ subject + sample type + treatment method + sample type * treatment method). After confirming sample type-treatment specific effects with the sample type * treatment method interaction term, analyses stratified by sample typewere used (feature ~ lyPMA + Benzonase + HostZERO + MolYsis + QIAamp, strata = subject id). To quantify the potential bias of treatment compared to controls, paired beta diversity indices between each treated and untreated sample were extracted and used for a subsequent linear mixed effects model.

The effect of treatment on alpha diversity and on species-specific differential abundance was identified by a linear mixed effect model implemented in lme4::lmer (feature ~ sample type + lyPMA + Benzonase + HostZERO + MolYsis + QIAamp + (1|subject id)). Analyses performed for differential abundance first implemented a centered log-ratio transformation to account for compositionality prior to regression modeling using *microbiome* R package v1.22.0 [38]. The false discovery rate (FDR) was calculated using the *qvalue* R package v2.32.0 [39].

Mediation analysis was conducted using *mediation* package v4.5.0 [40] with species richness as an outcome, the treatment method as an exposure, the final reads as a mediator, and sample type as a mediator-outcome confounders. Mixed effects linear regression was used for both outcome and mediator models, and the analysis was stratified by each treatment method.

All the data were visualized using R packages ggplot2 v3.4.4 [41] and ggpubr v0.6.0 (https://github.com/kassambara/ggpubr/)

For further identification of contaminants, two different statistical decontamination methods were employed. Firstly, the *decontam* package [23] was used with the *comb* ∈ *ed* method based on bacterial DNA from the qPCR result as total DNA concentration. The analysis employed negative controls, BAL, nasal, and sputum without negative controls after prevalence and abundance filtering. Secondly, we estimated decontaminated genus-level relative abundances using *t* ∈ *yvamp*[24] based on the MetaPhlAn read count table. The community compositions of the negative control and mock community samples were treated as known, and the relative abundance profiles were estimated for each of the remaining samples. A single contaminant relative abundance profile was estimated for each protocol. Detection efficiencies were estimated for taxa in the mock community relative to *E. faecalis*. The expected number of reads attributable to contamination was assumed to be inversely proportional to the bacterial DNA concentration in each sample (parameter *Z*-). Optimization of the unweighted Poisson criterion was performed until convergence was reached. Optimization was performed separately for each of the six protocols. Despite its presence in the mock community, *S. enterica* was not detected in any of the mock community samples in the HostZERO protocol. Therefore, to enable estimation, a pseudocount of 1 was imputed for a single positive control sample for this protocol only. For downstream analysis we only considered the estimated relative abundances of the samples of unknown composition (output matrix P).

## Supplementary Material

Supplement 1

## Figures and Tables

**Figure 1:**
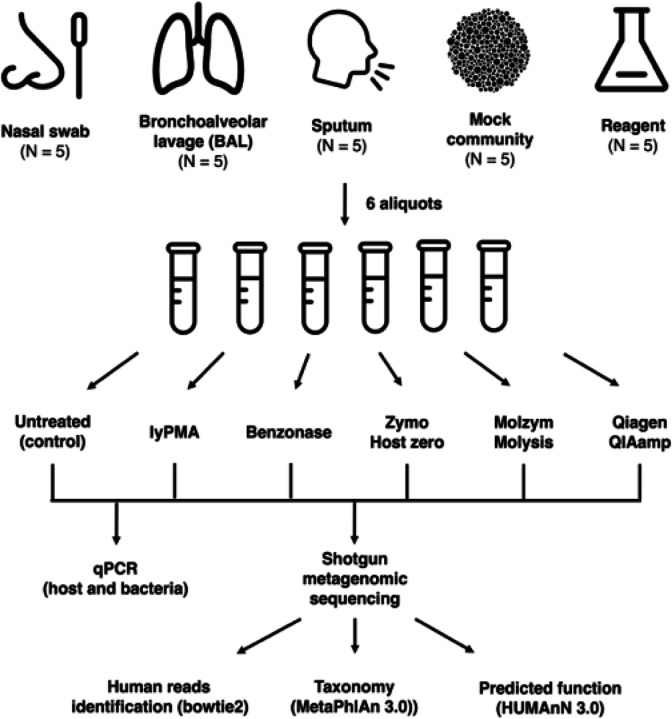
Overview of study design. Samples collected from the same participant were aliquoted so that paired comparisons could be made between treated and untreated samples. For nasal samples, it was only feasible to collect 4 swabs from a participant at the same time, thus a total of 10 swabs for the untreated condition was required to allow these paired treated and untreated comparisons.

**Table 1.** Summary statistics of host depletion treatment effect stratified by sample type and host depletion treatment. Number of samples in each experimental group, human and bacterial DNA quantified by qPCR, number of samples did not pass the library QC or failed sequencing (defined as no microbial mapped reads), QC’d read, % host mapped reads, final reads, average observed species richness, and predicted microbial functional richness. Values depicted as N (%) or median (interquartile range).

Sample	Treatment	N	Human DNA pg/µL	Bacterial DNA pg/µL	Sequencing fail^a^ N (%)	QC'd reads reads x 10^6^	Proportion host reads %	Final reads^b^ reads x 10^6^	Species richness	Function richness
BAL	Untreated	5	1,512.8 (1,237.9, 9,836.4)	12.6 (10.5, 37.8)	1^c^ (20 %)	129.5 (52.5, 129.9)	99.7 (99.6, 99.7)	0.33 (0.30, 0.43)	3 (1, 3)	6 (0, 8)
	lyPMA	5	2,139.7 (60.4, 6,255.6)	8.4 (0.3, 17.4)	2^c^ (40 %)	46.7 (28.6, 110.0)	99.1 (97.8, 99.5)	0.62 (0.44, 1.01)	6 (3, 7)	64 (14, 97)
	Benzonase	5	59.6 (47.8, 70.1)	0.9 (0.7, 2.3)	0 (0 %)	149.3 (129.7, 183.7)	98.8 (98.7, 98.9)	1.66 (1.61, 2.18)	6 (5, 7)	152 (107, 163)
	HostZERO	5	6.8 (2.3, 7.5)	0.4 (0.3, 1.1)	1 (20 %)	31.9 (18.4, 35.1)	83.7 (76.8, 87.2)	2.36 (1.33, 8.16)	8 (7, 11)	210 (119, 219)
	MolYsis	5	7.6 (6.6, 25.2)	2.0 (0.3, 4.6)	1 (20 %)	39.0 (29.0, 39.3)	92.5 (92.5, 93.6)	2.92 (1.27, 15.65)	18 (7, 45)	216 (212, 245)
	QIAamp	5	33.1 (32.0, 79.5)	0.5 (0.3, 1.9)	0 (0 %)	132.4 (119.6, 137.5)	98.3 (92.3, 98.6)	2.63 (0.96, 10.23)	8 (6, 14)	215 (30, 217)
Nasal	Untreated	10^d^	340.2 (202.3, 685.8)	22.9 (16.9, 26.6)	0 (0 %)	106.2 (63.7, 138.7)	94.1 (92.8, 97.9)	4.82 (1.05, 8.73)	12 (8.75, 12)	139 (115.75, 152)
	lyPMA	5	2.6 (0.8, 9.2)	0.3 (0.3, 0.3)	4 (80 %)	7.9 (6.9, 9.7)	91.2 (35.6, 91.6)	0.66 (0.58, 0.85)	5 (5, 7)	136 (123, 168)
	Benzonase	5	12.8 (1.9, 78.8)	6.1 (5.4, 10.2)	0 (0 %)	47.1 (41.7, 53.2)	78.7 (77.8, 94.8)	2.77 (2.59, 10.43)	8 (7, 14)	185 (124, 187)
	HostZERO	5	0.5 (0.1, 0.7)	7.6 (3.3, 15.8)	2 (40 %)	24.5 (11.7, 55.2)	8.9 (2.7, 30.4)	24.26 (9.74, 50.30)	19 (19, 20)	208 (195, 210)
	MolYsis	5	0.4 (0.0, 0.8)	1.6 (1.1, 5.8)	4 (80 %)	8.1 (5.0, 34.9)	49.9 (5.0, 78.4)	3.16 (1.75, 25.31)	11 (11, 21)	193 (183, 200)
	QIAamp	5	2.1 (0.9, 7.1)	28.8 (24.7, 30.7)	0 (0 %)	56.2 (54.9, 58.5)	20.1 (15.7, 23.2)	46.27 (45.02, 46.73)	18 (17, 20)	206 (188, 210)
Sputum	Untreated	5	39,231.5 (19,448.0, 59,430.9)	245.3 (220.3, 311.0)	0 (0 %)	69.2 (68.0, 75.6)	99.2 (98.9, 99.2)	0.60 (0.56, 0.88)	13 (10, 14)	149 (143, 150)
	lyPMA	5	9,779.5 (994.2, 11,437.5)	97.8 (25.9, 100.9)	0 (0 %)	89.7 (42.0, 105.2)	96.4 (92.5, 98.3)	2.48 (1.54, 4.42)	50 (42, 64)	251 (241, 257)
	Benzonase	5	154.3 (141.3, 349.0)	33.2 (21.3, 53.0)	0 (0 %)	84.0 (82.0, 87.1)	94.2 (92.9, 94.5)	4.71 (4.53, 5.93)	92 (84, 94)	270 (263, 302)
	HostZERO	5	49.4 (11.7, 57.7)	38.9 (33.6, 39.0)	0 (0 %)	106.2 (61.6, 114.8)	61.7 (37.5, 68.0)	29.07 (23.62, 36.72)	129 (110, 130)	307 (292, 320)
	MolYsis	5	13.6 (8.0, 26.3)	28.4 (24.1, 30.4)	0 (0 %)	105.6 (90.8, 115.7)	32.8 (17.0, 33.8)	61.06 (55.63, 83.68)	124 (121, 131)	309 (305, 313)
	QIAamp	5	241.6 (196.3, 273.5)	64.3 (34.6, 71.0)	0 (0 %)	102.4 (100.9, 106.0)	88.2 (68.9, 88.6)	11.63 (11.28, 38.86)	103 (88, 111)	276 (270, 277)

a Samples that failed library prep were still sequenced. Sequenced samples with 0 microbial mapped reads after QC and host read removal were considered to have failed sequencing.

b Final reads were calculated after removing samples that failed sequencing.

c Includes 1 sample failed in sequencing that showed zero species richness.

d Only 4 nasal could be collected from one individual at each time point thus with 6 treatment conditions, 4 nasal were collected from 10 participants and randomized to each treatment condition

**Table 2.** Overall effect of host depletion treatment based on statistical models. Changes were assessed using linear mixed effect models (features ~ treatment + (1|subject)) stratified by sample type. *: *p*-value < 0.05, **: *p*-value < 0.01 and ***: *p*-value < 0.001.

Sample type	Treatment	% Host	log_10_(Final reads)	Species richness	Functional richness	Bias (Morisita-Horn dissimilarities)	% gram negative	Comment
BAL	lyPMA	−3.1 (−15.7, 9.5)	0.4 (−0.2, 0.9)	1.8 (−10.3, 13.8)	58.6 (−45.1, 162.3)	0.3 (0.1, 0.6)*	6.4 (−21.2, 34.1)	Low efficiency
	Benzonase	−1.1 (−13.8, 11.5)	0.8 (0.3, 1.3)*	5.2 (−6.3, 16.8)	126.4 (22.7, 230.1)*	0.1 (−0.1, 0.3)	15.3 (−12.3, 43.0)	Low efficiency
	HostZERO	−18.3 (−30.9, −5.6)*	1.0 (0.4, 1.5)**	8.4 (−3.1, 20.0)	164.6 (60.9, 268.3)**	0.3 (0.0, 0.5)	8.6 (−19.0, 36.3)	No increased richness
	MolYsis	−17.7 (−30.3, −5.1)*	1.0 (0.5, 1.6)**	18.4 (6.9, 30.0)**	190.0 (86.3, 293.7)***	0.2 (0.0, 0.4)	3.4 (−24.3, 31.0)	Optimal
	QIAamp	−6.3 (−18.9, 6.3)	1.0 (0.5, 1.6)**	9.0 (−2.5, 20.6)	130.2 (26.5, 233.9)*	0.3 (0.0, 0.5)	7.3 (−20.4, 35.0)	Low efficiency
Nasal swabs	lyPMA	−27.7 (−49.0, −6.3)*	−0.5 (−1.0, −0.1)*	−4.5 (−8.9, −0.1)	9.1 (−23.2, 41.4)	0.2 (0.1, 0.3)*	19.4 (14.1, 24.6)***	No increased Richness
	Benzonase	−20.0 (−41.4, 1.5)	0.1 (−0.3, 0.6)	−0.1 (−4.5, 4.3)	22.5 (−9.8, 54.8)	0.2 (0.0, 0.3)*	1.9 (−3.4, 7.5)	Low Efficiency
	HostZERO	−73.6 (−94.9, −52.1)***	0.9 (0.4, 1.3)**	10.3 (5.9, 14.7)***	66.3 (34.0, 98.6)***	0.0 (−0.1, 0.2)	0.0 (−5.3, 5.6)	High rate Of Library Prep Failure
	MolYsis	−50.6 (−72.0, −29.3)***	0.2 (−0.2, 0.7)	6.3 (1.9, 10.7)*	53.5 (21.2, 85.8)**	0.2 (0.1, 0.3)**	2.3 (−3.0, 7.6)	High bias
	QIAamp	−75.4 (−96.9, −54.0)***	1.1 (0.6, 1.5)***	7.5 (3.1, 11.9)**	65.7 (33.4, 98.0)***	0.1 (0.0, 0.3)	0.1 (−5.4, 5.5)	Optimal
**Sputum**	lyPMA	−3.8 (−15.4, 7.8)	0.5 (0.3, 0.8)**	36.8 (18.9, 54.7)**	79.8 (16.5, 143.1)*	0.3 (0.1, 0.6)**	−40.9 (−52.6, −29.1)***	Low Efficiency
	Benzonase	−6.3 (−17.9, 5.4)	0.8 (0.6, 1.1)***	65.8 (47.9, 83.7)***	85.8 (22.5, 149.1)**	0.5 (0.3, 0.7)***	−52.5 (−64.2, −40.7)***	Low Efficiency
	HostZERO	−45.5 (−57.1, −33.8)***	1.7 (1.4, 1.9)***	101.8 (83.9, 119.7)***	137.0 (73.7, 200.3)***	0.6 (0.4, 0.8)***	−59.9 (−71.6, −48.1)***	Optimal^1^
	MolYsis	−69.6 (−81.3, −58.0)***	2.0 (1.7, 2.2)***	111.4 (93.5, 129.3)***	141.8 (78.5, 205.1)***	0.6 (0.4, 0.8)***	−59.9 (−71.6, −48.1)***	High bias^2^
	QIAamp	−18.7 (−30.3, −7.1)**	1.4 (1.2, 1.7)***	84.2 (66.3, 102.1)***	115.6 (52.3, 178.9)***	0.6 (0.4, 0.8)***	−60.6 (−72.3, −48.8)***	High bias

1 No bias measured at PERMANOVA

2 Higher bias measured at PERMANOVA

## Data Availability

All raw sequencing data is available under BioProject accession number PRJNA1019400 at the NCBI Sequencing Read Archive (SRA). Full documentation including data wrangling, exploratory data analyses, data processing, statistical modeling, and code for figure and table generation is available at a GitHub repository (https://github.com/minsiksudo/lai_lab_host_depletion).
