# Supplementary material for: Host DNA depletion on frozen human respiratory samples enables successful metagenomic sequencing for microbiome studies
Source: Res Sq. 2024 Jan 23:rs.3.rs-3638876. Preprint. [Version 1] doi: 10.21203/rs.3.rs-3638876/v1 (PMC10854296; doi:10.21203/rs.3.rs-3638876/v1)
Supplement: Supplement 1 [file NIHPPRS3638876v1-supplement-1.pdf]

Supplementary Files

This is a list of supplementary files associated with this preprint. Click to download.

- REP20231119MGKHOSTsupplementmicrobiome.docx
